# Effect of Tween 40 and DtsR1 on l-arginine overproduction in *Corynebacterium crenatum*

**DOI:** 10.1186/s12934-015-0310-9

**Published:** 2015-08-12

**Authors:** Minliang Chen, Xuelan Chen, Fang Wan, Bin Zhang, Jincong Chen, Yonghua Xiong

**Affiliations:** College of Life Science, Nanchang University, Nanchang, 330031 People’s Republic of China; Key Laboratory of Functional Small Organic Molecule, Ministry of Education, Jiangxi Normal University, 99 Ziyang Road, Nanchang, 330022 People’s Republic of China; State Key Laboratory of Food Science and Technology, Jiangxi-OAI Joint Research Institute, Nanchang University, 235 Nanjing East Road, Nanchang, 330047 People’s Republic of China

**Keywords:** *Corynebacterium crenatum*, l-Arginine, Tween 40, DtsR1, α-Oxoglutarate dehydrogenase complex, NADP^+^/NADPH

## Abstract

**Background:**

l-Glutamate is an important precursor in the l-arginine (l-Arg) biosynthetic pathway. Various methods, including polyoxyethylene sorbitan monopalmitate (Tween 40) addition and *dtsR1* disruption, have been widely used to induce l-glutamate overproduction in *Corynebacterium glutamicum*. In this study, a novel strategy for l-Arg overproduction through Tween 40 trigger and Δ*dtsR1* mutant were proposed in *Corynebacterium crenatum*.

**Results:**

*Corynebacterium crenatum* mutant (CCM01) was selected as a host strain, whose *argR* was lethal via mutagenesis screening, the *proB* gene was knocked out, and *argB* was replaced by *argB* M4 (E19R, H26E, D311R, and D312R) to release l-Arg feedback resistance. After Tween 40 trigger in the logarithmic period, l-Arg production increased from 15.22 to 17.73 g/L in CCM01 strain. When *NCgl1221* and *dtsR1* disruption (CCM03), l-Arg production drastically increased to 27.45 g/L and then further to 29.97 g/L after Tween 40 trigger. Moreover, the specific activity of α-oxoglutarate dehydrogenase complex (ODHC) decreased, whereas the regeneration of NADP^+^/NADPH significantly increased after *dtsR1* disruption and Tween 40 trigger. Results of real-time PCR showed that the transcriptional levels of *odhA*, *sucB*, and *lpdA* (encoding three subunits of the ODHC complex) were downregulated after Tween 40 trigger or *dtsR1* disruption. By contrast, *zwf* transcription (encoding glucose-6-phosphate dehydrogenase) showed no significant difference among CCM01, CCM02 (Δ*NCgl1221*), and CCM03 (Δ*NCgl1221*Δ*dtsR1*) strains without Tween 40 trigger but evidently increased by 5.50 folds after Tween 40 trigger.

**Conclusion:**

A novel strategy for l-Arg overproduction by *dtsR1* disruption and Tween 40 trigger in *C. crenatum* was reported. Tween 40 addition exhibited a bifunctional mechanism for l-Arg overproduction, including reduced ODHC activity and enhanced NADPH pools accumulation by downregulated *dtsR1* expression and upregulated *zwf* expression, respectively.

## Background

l-Arginine (l-Arg) is a semi-essential amino acid that is widely used as an additive in food, cosmetic, and pharmaceutical industries because of its ability to promote secretion of growth hormones [1], insulin [2], and prolactin [3] and facilitate the synthesis of various immune active factors to prevent cancer cell growth [4]; l-Arg is a nitric oxide precursor for relaxing and dilating blood vessels [5]. Different microorganisms, such as *Corynebacterium glutamicum* [6, 7], *Escherichia coli* [8], *Bacillus subtilis* [9], and *Saccharomyces cerevisiae* [10], are used as model organisms for l-Arg overproduction.

**Fig. 1:**
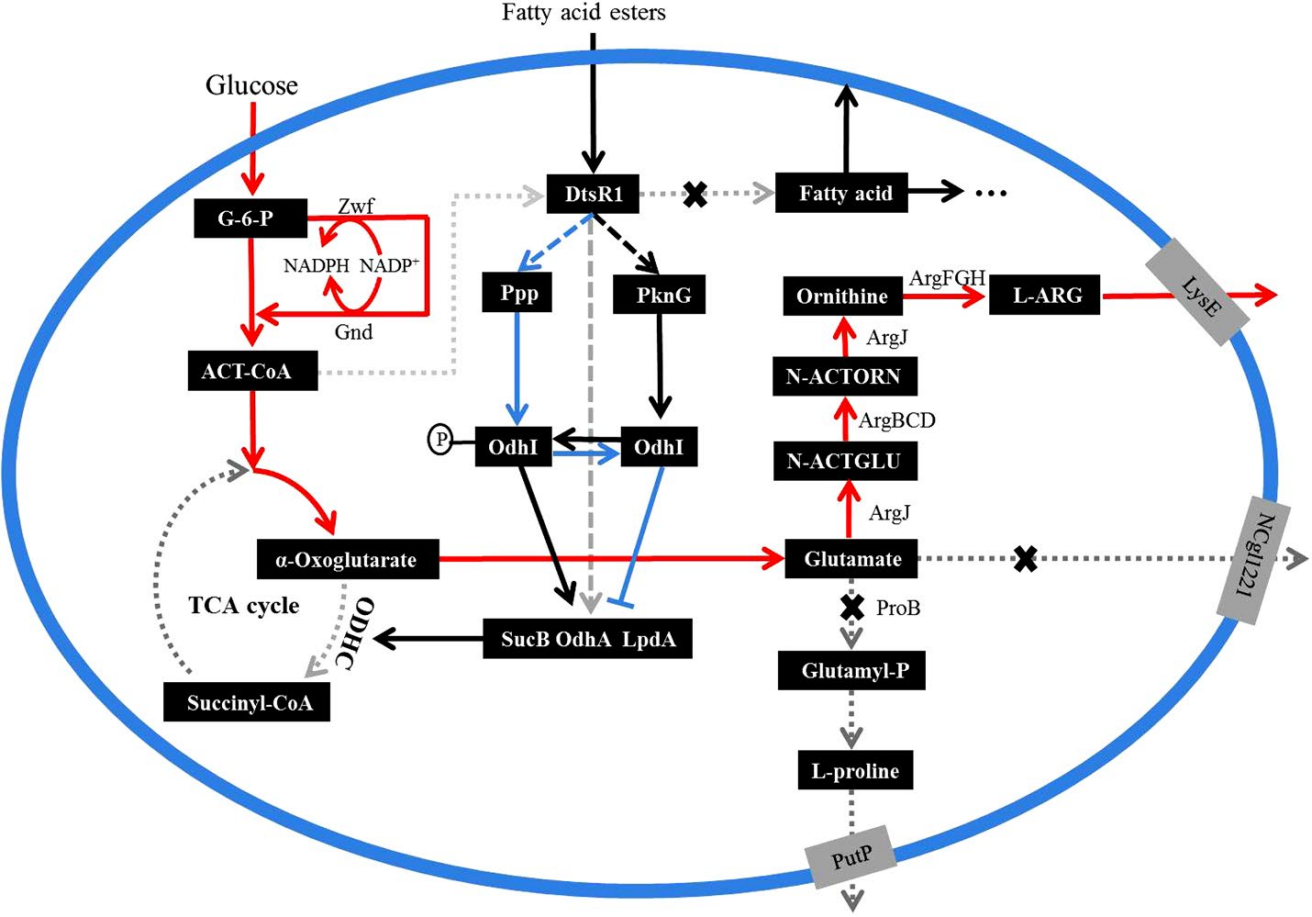
Correlation biosynthetic pathway of l-Arg and possible signaling cascade involved in the regulation of ODHC specific activity under Tween 40 addition by *C. crenatum* (Brief). *Red thick arrows* indicate increased fluxes by overexpressing the corresponding genes. *Dot-dashed lines* represent decreased or cut off fluxes by reducing relevant enzyme activity and knocking out relevant genes, respectively. *G-6-P* glucose-6-phosphate, *ACT-CoA* acetyl-CoA, *Zwf* glucose-6-phosphate dehydrogenase, *Gnd* 6-phosphogluconate dehydrogenase, *ProB* gamma-glutamyl kinase, *PutP* l-proline exporter, *NCgl1221* glutamate exporter, *LysE* l-Arg and l-lysine exporter, *N-ACTGLU* *N*-acetylglutamate, *N-ACTORN* *N*-acetylornithine, *argJBCDF*-*argGH*, the gene clusters of l-Arg biosynthetic pathway, *DtsR1* acetyl-CoA carboxylase, *AccBc* biotin carboxylase and biotin carboxyl carrier protein, *PknG* serine/threonine protein kinase, *Ppp* protein phosphatase, *OdhI* signal transduction protein, FHA-domain-containing protein, *OdhA* α-oxoglutarate dehydrogenase, *LpdA* dihydrolipoamide dehydrogenase, *SucB* dihydrolipoamide acetyltransferase.

In recent decades, various strategies based on genetic engineering technology have been designed to improve industrial levels of l-Arg. Ginesy et al. [8] reported an engineered *E. coli* strain for l-Arg overproduction by deleting the *speC*, *speF*, *argA*, *adiA*, and *argR* genes, introducing feedback-resistant *argA214*, and overexpressing *argO* genes, whose l-Arg final production was achieved at 11.64 g/L in 1-L batch fermentation. Xu et al. [11] also performed site-directed mutagenesis of *N*-acetyl-l-glutamate kinase (including E19R, H26E, and H268D) to alleviate feedback inhibition by l-Arg; l-Arg production improved by about 41.7 % as compared with that of the initial strain. Moreover, Xu et al. overexpressed l-Arg biosynthetic genes, including the *arg*C ~ H cluster [12] and the *lysE* gene [13] in *Corynebacterium crenatum* SYPA 5–5; l-Arg production was achieved at 45.3 and 35.9 g/L, respectively, by batch fermentation in a 5-L bioreactor. Lee et al. [14, 15] reported a metabolically engineered *C. glutamicum* for production of l-Arg at the industrial-scale based on systems metabolic engineering, including random mutagenesis to release feedback inhibition, knocking out *argR* and *farR* genes to remove repressors, increasing NADPH and carbamoyl phosphate pools, and deleting *NCgl1221* gene to avoid l-glutamate exporter. Subsequently, l-Arg production distinctly increased to 92.5 g/L by fed-batch fermentation in a 5-L bioreactor.

l-Glutamate is an important precursor in the l-Arg biosynthetic pathway. Previous research has demonstrated that reduced α-oxoglutarate dehydrogenase complex (ODHC) activity can significantly induce l-glutamate overproduction in *C. glutamicum* by increasing metabolic fluxes toward l-glutamate synthesis (as shown in Fig. 1). As a fermentation trigger, polyoxyethylene sorbitan monopalmitate (Tween 40) is widely used to enhance l-glutamate overproduction in *C. glutamicum* because it can decrease ODHC activity by downregulation of *dtsR1* expression [16]. DtsR1 protein is a homolog of the subunit of the biotin enzyme acetyl-CoA carboxylase complex, and disruption of the *dtsR1* gene can notably reduce ODHC activity, leading to an increase in l-glutamate production [17, 18]. However, to our knowledge, the strategy by adding Tween 40 and deleting *dtsR1* for l-Arg overproduction has not been published. *C. crenatum* is highly homologous to *C. glutamicum* and is frequently used for production of various amino acids, including l-Arg, because of its genetic tractability, bio-safety, and robustness in fermentation [19, 20]. In this work, *C. crenatumargB*-M4 Δ*proB*, whose *argR* was lethal via mutagenesis screening, *proB* gene was deleted, and *argB* gene was replaced by *C. crenatumargB* M4 gene (E19R, H26E, D311R, and D312R) to release l-Arg feedback inhibition, was chosen as a host strain to study l-Arg production. Furthermore, *NCgl1221* (encoded as l-glutamate exporter) and *dtsR1* genes were knocked out using a marker-free system to increase l-Arg precursor. l-Arg production, ODHC specific activity, and NADPH pools accumulation by Tween 40 trigger and *dtsR1* gene regulation were explored, and the related genes transcription in response to inducing l-Arg production was investigated to elaborate the relationship among *dtsR1* gene deletion, Tween 40 addition, and l-Arg overproduction.

## Results and discussion

### Effect of Tween 40 and DtsR1 on l-Arg production in *C. crenatum*

**Fig. 2:**
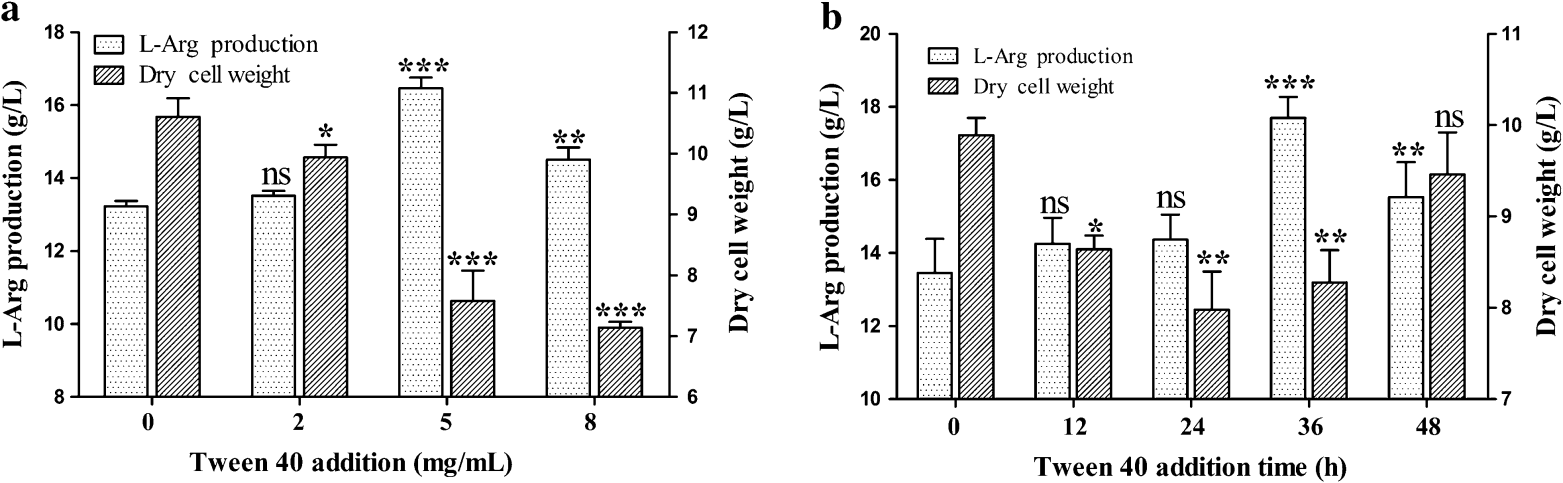
Effect of Tween 40 addition (0–8 mg/mL Tween 40, after 36 h incubation time) and addition time (0–48 h, 5 mg/mL Tween 40) on l-Arg production in CCM01 strain. l-Arg production and the DCW were monitored during shake flask cultivation for 120 h by Tween 40 addition. Results are the means ± standard deviations from three independent experiments. Compared with the control group, “ns” designates *P* > 0.05, *P < 0.05; **P < 0.01; ***P < 0.001.

**Fig. 3:**
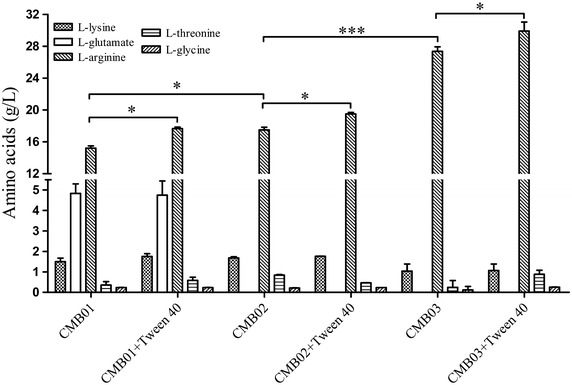
Concentrations of various amino acids of different *C. crenatum* strains cultured at 30 °C with Tween 40 addition in shake flask cultivation. Results are the means standard deviations from at least three independent experiments. *P < 0.05; ***P < 0.001.

Tween 40, as a fermentation trigger, can induce l-glutamate overproduction in *C. glutamicum* [21, 22]. To elaborate the effect of Tween 40 on l-Arg production in *C. crenatum*, we optimized Tween 40 addition and addition time in CCM01 strain. As shown in Fig. 2a, the biomass of *C. crenatum* gradually decreased with increasing concentration of Tween 40 from 0 to 8.0 mg/mL, and the l-Arg production reached a maximum value when Tween 40 concentration was 5 mg/mL in the fermentation medium. The addition time of Tween 40 on l-Arg production is shown in Fig. 2b, indicating that l-Arg overproduction could not be induced by Tween 40 addition before the 24 h fermentation time. During the logarithmic period (after 36 h fermentation time), Tween 40 addition significantly increased l-Arg production from 15.22 to 17.73 g/L (*P* < 0.05), while the yield of l-Arg per gram biomass was increased from 1.54 to 2.24 g/g. Moreover, the yield of l-Arg per gram glucose increased from 0.22 to 0.30 g/g, consequently increasing l-Arg productivity from 0.13 to 0.15 g/L/h in CCM01 strain (Table 1). The above results indicate that adding Tween 40 at the logarithmic period is conducive to l-Arg production. Meanwhile, the concentrations of other amino acids were analyzed using an amino acid analyzer. The results shown in Fig. 3 indicated that a large amount of l-glutamate was secreted into the fermentation supernatant (4.72 g/L). To avoid l-glutamate leak during l-Arg fermentation, *NCgl1221* gene encoding l-glutamate transporter [23, 24] was removed from the genome of CCM01 strain (named CCM02) to promote l-Arg production. As expected, extracellular l-glutamate in CCM02 strain decreased to an undetectable level (Fig. 3), whereas l-Arg production increased to 19.56 g/L after Tween 40 trigger. Although Tween 40 addition increased l-Arg production in *C. crenatum*, the increased yield of l-Arg is very limited. A previous study has confirmed that disruption of *dtsR1* can convert the metabolic fluxes from tricarboxylic acid cycle toward glutamate synthesis by repressing ODHC specific activity [18]. To further improve l-Arg production, we knocked out the *dtsR1* gene in CCM02 strain (named CCM03). The results showed that extracellular l-Arg production in CCM03 strain drastically increased to 27.45 g/L, whereas l-lysine production evidently declined by 39.56 % to 1.06 g/L compared with 1.75 g/L in CCM01 + Tween 40 condition (Fig. 3). The yield of l-Arg per gram glucose increased to 0.34 g/g, improving by 54.55 % as compared with that in CCM01 strain (Table 1). Although l-Arg production after DtsR1 disruption is still low compared with the highest level (92.5 g/L) of that reported by Lee et al. [14, 15], it is notable that the yield of l-Arg per gram glucose is basically consistent with the previous report, suggesting the CCM03 strain exhibits great potential to improve the yield of l-Arg by fed-batch fermentation. In addition, l-Arg production could further improve to approximately 9.12 % (29.97 g/L) in CCM03 strain after Tween 40 addition. To further elaborate the function of Tween 40 and DtsR1 protein on l-Arg overproduction in *C. crenatum*, we investigated ODHC specific activity and NADPH pools accumulation.

**Table 1:** Comparison of the performances of the different conditions for l-Arg production by fermentation

Conditions	GlcC (g/L)	DCW (g/L)	Arginine (g/L)	Yield		V_P_ (g/L/h)
				Y_A/G_ (g arg/g glc)	Y_A/D_ (g arg/g DCW)	
CCM01	70.32 ± 3.35	9.92 ± 0.35	15.22 ± 0.27	0.22 ± 0.01	1.54 ± 0.01	0.13 ± 0.03
CCM01 + Tween 40	60.33 ± 2.47	7.91 ± 0.42	17.73 ± 0.18	0.30 ± 0.02	2.24 ± 0.03	0.15 ± 0.04
CCM02	60.96 ± 1.05	8.34 ± 1.07	17.58 ± 0.34	0.29 ± 0.04	2.11 ± 0.02	0.15 ± 0.04
CCM02 + Tween 40	59.37 ± 2.91	7.36 ± 0.27	19.56 ± 0.19	0.33 ± 0.03	2.68 ± 0.02	0.16 ± 0.02
CCM03	80.98 ± 1.05	8.17 ± 1.07	27.45 ± 0.58	0.34 ± 0.05	3.38 ± 0.13	0.23 ± 0.02
CCM03 + Tween 40	89.34 ± 3.91	7.36 ± 0.27	29.97 ± 1.13	0.34 ± 0.09	4.10 ± 0.15	0.25 ± 0.05

Fermentations were performed in a 250 mL flask at 30 °C; the initial glucose concentration was 120 g/L.

Results are the means ± standard deviations in three independent experiments.

*GlcC* glucose consumed, *Y*_*A/G*_ arginine yield vs. glucose, *Y*_*A/D*_ arginine yield vs. DCW, *V*_*P*_ volumetric productivity.

### Effect of Tween 40 and DtsR1 on ODHC specific activity and NADPH pools accumulation in *C. crenatum*

**Fig. 4:**
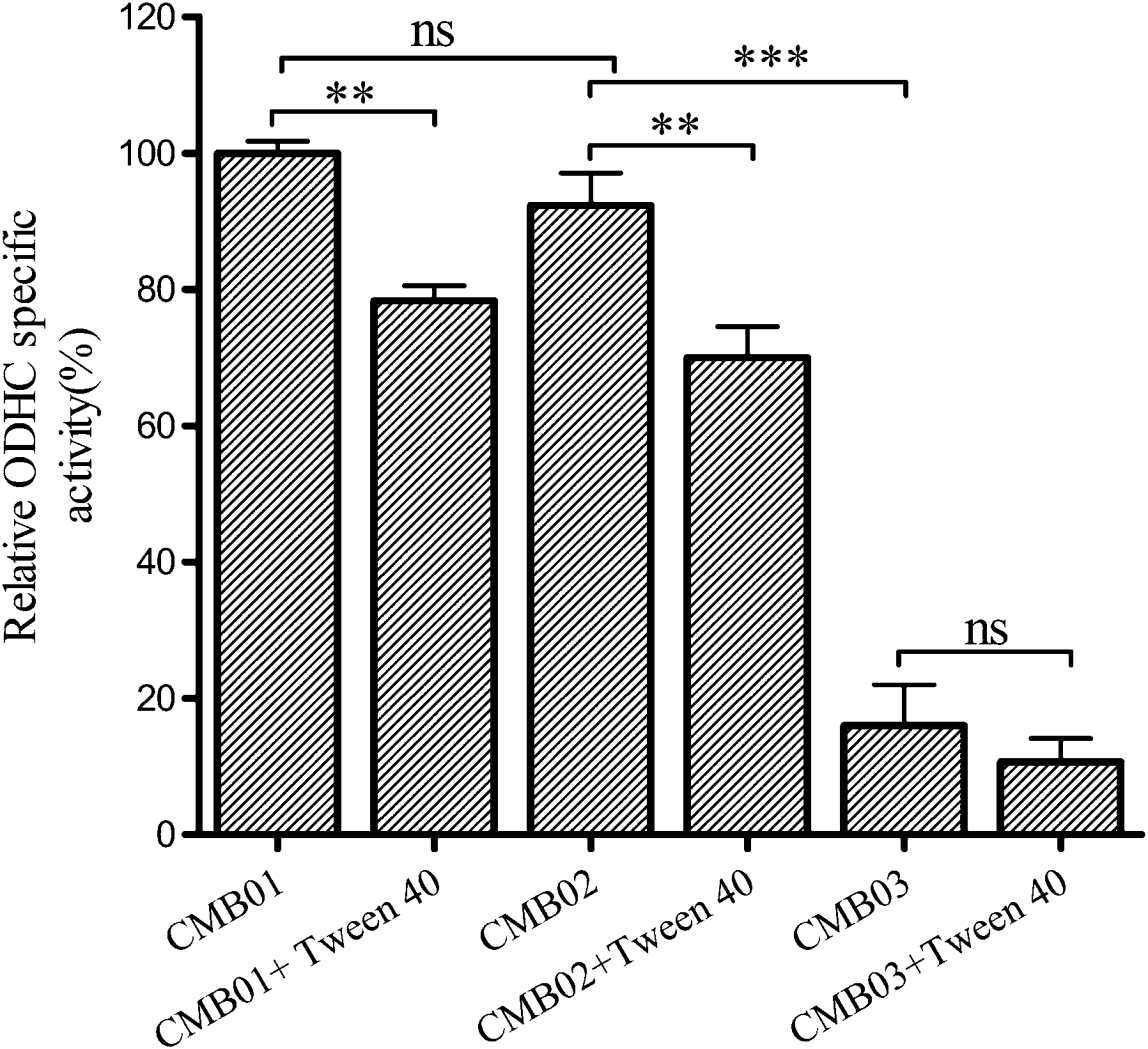
Relative ODHC specific activity of the different strains under Tween 40-triggered l-Arg production in shake flask cultivation. Results are the means ± standard deviations from three independent experiments. Compared with the control group, “ns” designates *P* > 0.05, **P < 0.01, ***P < 0.001.

ODHC is a branch-point enzyme complex between the tricarboxylic acid cycle and l-glutamate biosynthesis. Tween 40 addition can indirectly convert the metabolic fluxes into the l-glutamate biosynthetic pathway by downregulating *dtsR1* gene expression; the disruption of *dtsR1* gene can reduce the ODHC activity, thereby inducing l-glutamate overproduction in *C. glutamicum*. In addition, l-glutamate production induced by Tween 40 addition can completely be suppressed by *dtsR1* gene disruption [25]. In the present study, ODHC specific activity decreased by 20.23 and 36.67 % in CCM01 and CCM02 strains, respectively, during l-Arg fermentation after Tween 40 trigger (as shown in Fig. 4). When *dtsR1* gene was removed from the genome of *C. crenatum* (CCM03 strain), ODHC specific activity decreased to about 82.56 % compared with that in CCM01 strain, and ODHC specific activity did not further decline after Tween 40 addition (*P* > 0.05), which was consistent with a previous report [25]. However, l-Arg production further increased in CCM03 + Tween 40 condition (*P* < 0.05, Fig. 3). The above results strongly suggest that probably other mechanisms exist for l-Arg overproduction induced by Tween 40 trigger after *dtsR1* disruption in *C. crenatum*.

A recent study has confirmed that NADPH pools accumulation is also conducive to l-Arg production because the biosynthesis of 1 mol of l-Arg requires 3 mol of NADPH [14, 26]. DtsR1 protein is one of the subunits of acetyl-CoA carboxylase complex, which is involved in fatty acid biosynthesis. Fatty acid biosynthesis requires a large amount of NADPH pools for fatty acid elongation cycle [27]. After DtsR1 disruption, the intracellular level of NADPH notably increased to 0.59 mmol/L in CCM03 strain, which was approximately three times higher than that of CCM01 strain (Table 2). The above results suggest that a large amount of NADPH pools for fatty acid biosynthesis was converted for l-Arg biosynthesis. We also found that the NADPH level could further increase upon Tween 40 addition in CCM01, CCM02, and CCM03 strains. To date, relevant research about Tween 40 as a trigger for NADPH pools accumulation has not been reported.

**Table 2:** Intracellular NADP+ and NADPH concentrations in different conditions

Conditions	NADPH (mmol/L)	NADP^+^ (mmol/L)	NADP^+^/NADPH
CCM01	0.13 ± 0.01	0.25 ± 0.02	1.89
CCM01 + Tween 40	0.30 ± 0.04	0.45 ± 0.02	1.49
CCM02	0.15 ± 0.03	0.28 ± 0.04	1.90
CCM02 + Tween 40	0.45 ± 0.02	0.68 ± 0.05	1.51
CCM03	0.59 ± 0.03	0.78 ± 0.01	1.32
CCM03 + Tween 40	0.75 ± 0.18	0.98 ± 0.05	1.31

Results are the means ± standard deviations in three independent experiments.

### Regulation mechanism of Tween 40 and DtsR1 on l-Arg overproduction

As shown in Fig. 4 and Table 2, l-Arg overproduction by Tween 40 trigger or DtsR1 disruption is attributed to reduced ODHC enzymatic activity and NADPH pools accumulation. ODHC complex consists of three subunits, including α-oxoglutarate dehydrogenase encoded by *odhA* gene [28], dihydrolipoamide *S*-succinyltransferase encoded by *sucB* gene, and dihydrolipoamide dehydrogenase encoded by *lpdA* gene [29]. A recent study has confirmed that ODHC activity regulation is not only related to the expression level of DtsR1 protein [17] but also with the phosphorylated and unphosphorylated forms of OdhI protein [30, 31]. The downregulated expression of DtsR1 protein is in favor of reducing ODHC activity, and unphosphorylated OdhI is also as an inhibitor of ODHC because the FDH domain of unphosphorylated OdhI can combine with the OdhA subunit inhibiting ODHC activity [32]. OdhI protein can be phosphorylated by serine/threonine protein kinases, including PknG and PknB, and dephosphorylated by phosphoserine/threonine protein phosphatase Ppp [33, 34]. In addition, intracellular NADPH is mainly generated from the pentose phosphate pathway, in which the gene cluster of *tkt*–*tal*–*zwf*–*opcA*–*pgl* is involved. Among them, glucose-6-phosphate dehydrogenase (encoded by *zwf* gene) is responsible for regeneration of NADP^+^ to NADPH [35]. To elucidate the regulation mechanism of Tween 40 and DtsR1 for l-Arg overproduction, we investigated the transcriptional levels of *dtsR1*, *ppp*, *pknG*, *odhA*, *sucB*, *lpdA*, *zwf*, and *argB*, which is involved in the arginine biosynthetic pathway and is located in the gene cluster of *argCJBDF*-*argGH*, by real-time quantitative PCR method.

**Fig. 5:**
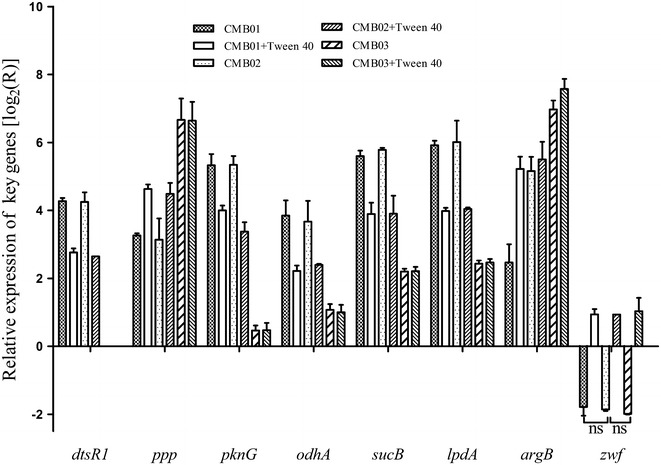
Relative transcription level of genes possible involved in the regulation of ODHC specific activity and of genes involved in the biosynthetic pathway of l-Arg triggered by Tween 40 addition and *dtsR1* disruption. Results are the means ± standard deviations from three independent experiments. Compared with the control group, “ns” designates *P* < 0.05.

The results shown in Fig. 5 indicate that *dtsR1* and *pknG* were downregulated by 3.12- and 3.14-fold, respectively, whereas *ppp* was upregulated by 2.80-fold in CCM01/02 strain after Tween 40 trigger, and the transcriptional levels of *odhA*, *sucB*, and *lpdA* were concomitantly downregulated by 3.20-, 2.47-, and 2.19-fold, respectively, which were consistent with the previous report by Kataoka et al. [36]. We also found that the expression level of *argB* was upregulated by 17.25-fold after removing the *NCgl1221* gene in CCM02 strain. We suspected that the high expression of *argB* gene is associated with intracellular l-glutamate accumulation. After *dtsR1* disruption, the transcriptional level of *pknG* notably downregulated by 6.00-fold, and *ppp* transcription was upregulated by 8.53-fold, which resulted in lesser transcriptional levels of *odhA*, *sucB*, and *lpdA* in CCM03 strain compared with those in CCM01 strain. Meanwhile, the expression levels of *pknG*, *ppp*, *odhA*, *sucB*, and *lpdA* remained unaltered in CCM03 strain after Tween 40 trigger. The above results demonstrated that reducing ODHC enzymatic activity induced by Tween 40 could be completely suppressed by *dtsR1* disruption in *C. crenatum*. We also found that *zwf* transcription showed no significant difference among CCM01, CCM02, and CCM03 strains (*P* > 0.05). The above results indicated that *NCgl1221* and *dtsR1* expression were not associated with NADP^+^/NADPH regeneration, and the increase in intracellular NADPH level after DtsR1 deletion could come from fatty acid synthesis. However, after adding Tween 40, the transcriptional level of *zwf* gene increased by 5.50-fold in CCM01, CCM02, and CCM03 strains. This result suggests that Tween 40 could be a key trigger in inducing NADPH regeneration for l-Arg overproduction.

## Conclusion

A novel strategy for overproducing l-Arg by reduction of ODHC enzymatic activity and promotion of NADPH accumulation in *C. crenatum* was reported. To avoid intracellular l-glutamate secretion and reduce ODHC specific activity, we successfully deleted *NCgl1221* and *dtsR1* by using a marker-free system in *C. crenatum*. l-Arg production significantly increased from 15.22 to 27.45 g/L, and l-Arg production further enhanced by 29.97 g/L after Tween 40 trigger. We also, for the first time, found that Tween 40 addition not only downregulated *dtsR1* expression but also induced NADP^+^/NADPH regeneration by upregulating *zwf* expression. However, further experiments, such as transcriptomics, proteomics, and metabolic engineering, are necessary to elucidate the more detailed mechanism of l-Arg overproduction by Tween 40 trigger.

## Methods

### Strains, plasmids and primers

*Corynebacterium crenatum argB*-M4 Δ*proB* strain (named CCM01) used as a parent strain in this study was constructed in our previous work (data not shown), whose *argR* was lethal via mutagenesis screening, the *proB* gene was knocked out, and *argB* was replaced by *argB* M4 (E19R, H26E, D311R, and D312R) to release l-Arg feedback resistance. The strains and plasmids used in this study are listed in Table 3. The primers used for strain construction and RT-quantitative (q) PCR amplification are listed in Table 4.

**Table 3:** Strains and plasmids used in this study

Strains/plasmids	Function and relevant characteristics	Sources
Strains		
DH5α	General clone host strain	Invitrogen
*C. crenatum* MT	Mutation strain with auxotrophic for biotin, and producing l-Arg	Lab stock
CCM01	*C. crenatum* MT with *proB* gene deletion, which E-*argB* gene replaced by *C. crenatum* *argB* M4 gene (E19R, H26E, D311R, and D312R)	This work
CCM02	Chromosomal deletion of the *Ncgl1221* gene of CCM01	This work
CCM03	Chromosomal deletion of the *dtsR1* gene of CCM02	This work
Plasmids		
pK18*mobsacB*	Mobilizable vector, allows for selection of double crossover in *C. crenatum*, Km^R^, *sacB*	Lab stock
pK18-Δ*NCgl1221*	A derivative of pK18*mobsacB*, harboring Δ*NCgl1221* fragment	This work
pK18-Δ*dtsR1*	A derivative of pK18*mobsacB*, harboring Δ*dtsR1* fragment	This work

**Table 4:** Sequences of oligonucleotide primers

Primers	Sequence (5′–3′)	Purposes
*NCgl1221*-up-F	CGC**AAGCTT**CAAGAAAGCCCTCGTTCCAACACTG	Amplifying the left arm of *NCgl1221*
*NCgl1221*-up-R	ATCAGCGTCCTAAGGAATCAAAAACGCCAAGACCAGG	
*NCgl1221*-down-F	GGCGTTTTTGATTCCCTTAGGACGCTGATTACAGACG	Amplifying the right arm of *NCgl1221*
*NCgl1221*-down-R	GCA**TCTAGA**GGAAGGGAGTTGAAGGTGACG	
*dtsR1*-up-F	CGC**AAGCTT**CAGCAAGTCAGCATTAGTGGAGC	Amplifying the left arm of *dtsR1*
*dtsR1*-up-R	GCCGATTTACAGTGTGAAATCGTAGCGG *TAGG*	
*dtsR1*-down-F	CCGCTACGATTTCACACTGTAAATCGGCGAATCC	Amplifying the right arm of *dtsR1*
*dtsR1*-down-R	GCA**TCTAGA**GGAAGGGAGTTGAAGGTGACG	
*odhA*-F	TCATTGAGGCATACCGCTCC	RP of *odhA*
*odhA*-R	TGAAGGTACGGTCCAGATCC	
*lpdA*-F	TGGGACTTAACCGTGGGCT	RP of *lpdA*
*lpdA*-R	CACACCGTTAATCATGCGGAC	
*sucB*-F	TCGTGAAGCGTCCAGTTGTC	RP of *sucB*
*sucB*-R	GGTCCTTGATGGTGGTCAGG	
*pknG*-F	GGCGGCATGGGTTGGATTT	RP of *pknG*
*pknG*-R	GTGCCTTGGTCTTGAACGGA	
*ppp*-F	CGGTATTGATCGTAGCCCTG	RP of *ppp*
*ppp*-R	CGCGACGTAAAAAGTGCTG	
*dtsR1*-F	CCTCCCATCCAACAATCGCT	RP of *dtsR1*
*dtsR1*-R	TAAGGAACGGTCGCGGAATC	
*zwf*-F	ACTGAGATTGCCGTGGTGTT	RP of *zwf*
*zwf*-R	AGCGGATGAGCACACCTTC	
*argB*-F	GTCGCGATTTAGTTGGTTTGAT	RP of *argB*
*argB*-R	GAGGCATCGACATTAATGATGTCT	
*16s rRNA*-F	AAGAAGCACCGGCTAACTAC	Reference Gene
*16s rRNA*-R	CCGGGATTTCACAGACGAC	

Restriction sites were highlighted in bold; linker sequences for crossover PCR were shown in underline.

*RP* RT-qPCR.

### Strain construction

*Escherichia coli* DH5α strain was used as a primary host for all gene cloning. *Corynebacterium crenatum* was grown in Luria–Bertani (LB) medium at 210 rpm and 30 °C, while *E. coli* was grown in LB medium at 180 rpm and 37 °C. Unless otherwise indicated, the concentration of Kanamycin was 25 μg/mL. CCM01 with deleted *NCgl1221* gene (named CCM02) was obtained by knocking out *NCgl1221* gene by using a marker-free system with *Bacillus subtilissacB* gene via two rounds of recombination as described previously [37]. The fusion arms containing 5′-upstream and 3′-downstream fragments were obtained by overlapping PCR. These fusion arms were then used for construction of recombinant plasmid by inserting them into the pK18*mobsacB* vector. The resultant recombinant plasmid was transferred into *C. crenatum* by electroporation. The single crossover strains were selected on LBHIS (LB supplemented with brain heart infusion and sorbitol) agar plates containing 10 μg/mL kanamycin, whereas marker-free recombinants were screened on sucrose-resistant agar plates. Moreover, CCM02 with deleted *dtsR1* gene (named CCM03) underwent the same procedure as the previous strains.

### l-Arg fermentation

The seed medium for *C. crenatum* cultivation (per liter) consisted of 30 g of glucose, 20 g of corn steep liquor, 20 g of (NH_4_)_2_SO_4_, 1 g of KH_2_PO_4_, 0.5 g of MgSO_4_·7H_2_O, and 1.5 g of urea. For seed culture, cells on the LB medium plate were inoculated into a test tube containing 5 mL of the LB medium, and cultivated in a shaking incubator with 210 rpm at 30 °C for 24 h. One milliliter of the culture was transferred to a 250 mL flask containing 30 mL of the seed medium. After the optical density of seed culture (OD_562_) was reached between 5.5 and 6.0, 2.0 mL seed culture was transferred into a 250 mL flask containing 25 mL the fermentation medium and cultured at 30 °C for 120 h. The fermentation medium (per liter) for l-Arg production was composed of 120 g of glucose, 25 g of corn steep liquor, 45 g of (NH_4_)_2_SO_4_, 1 g of KH_2_PO_4_, 0.5 g of MgSO_4_·7H_2_O, and 30 g of CaCO_3_ (pH 7.0). d-Biotin was supplied from the corn steep liquor of the medium [20]. For the growth of CCM03 strain (Δ*proB*Δ*NCgl1221*Δ*dtsR1*), l-proline and oleic acid ester (Tween 80) were added to media with final concentrations of 2.5 and 1 mg/mL, respectively. Tween 40 was added to the medium to a final concentration of 5 mg/mL once cell growth reached the early exponential phase to further trigger l-Arg overproduction. Cell concentration was determined at 562 nm and measured by a pre-calibrated relationship (1 OD = 0.375 g/L). 3,5-Dinitrosalicylic acid colorimetry was used to measure glucose concentration in the culture [38]. Concentrations of l-Arg and other amino acids were determined using a Sykam S-433D amino acid analyzer (Sykam Co. Ltd., Germany). All the data are presented as means ± standard deviations in three independent experiments.

### Measurement of ODHC specific activity and concentrations of intracellular NADP^+^ and NADPH pools

Samples preparation and ODHC specific activity were conducted according to a previously described method [39, 40]. According to the previous work, the l-Arg production rate was kept highest during 36–60 h fermentation time (data not show), therefore, *C. crenatum* cells were harvested in the late exponential phase (after 48 h cultivation), and collected by centrifugation at 5,000 rpm at 4 °C for 10 min. The pellets were diluted with 0.2 M HCl to dissolve CaCO_3_ and then washed twice with 0.2 % KCl solution. The pure cells were suspended in 5 mL of 0.1 M *N*-tris(hydroxymethyl)methyl-2-aminoethanesulfonic acid (TES)·NaOH buffer (pH 7.7) containing 30 % (v/v) glycerol and 10 mg/mL lysozyme. Following incubation at 37 °C for 3 h, the cells were disrupted by sonication and centrifuged to remove cell debris. The supernatant was collected, and the protein concentration was determined by Nanodrop 2000 (Thermo Scientific, Germany). ODHC specific activity assay was performed by adding 100 μL of cell extract to 2.5 mL of reaction mixture. The reaction mixture contained 100 mM TES·NaOH buffer (pH 7.7), 5 mM MgCl_2_, 3 mM cysteine, 0.3 mM thiamine pyrophosphate, 0.2 mM coenzyme A, and 1 mM 3-acetylpyridine adenine dinucleotide (APAD^+^). Upon adding 1 mM α-oxoglutarate to the reaction mixture, the initial increase in the absorbance of APADH at 365 nm was consecutively measured at 31.5 °C for 5 min with 30 s intervals. ODHC specific activity is defined as the amount of enzyme required to generate 1 µmol NADH per minute. The molar extinction coefficient of NAD^+^ was 9.1.

Intracellular NADP^+^ and NADPH concentrations were determined by enzymatic cycling reaction initiated with the EnzyChrom™ NADP^+^/NADPH Assay kit (BioAssay Systems, Hayward, CA 94545, USA). All the data are presented as the means ± standard deviations in three independent experiments.

### Real-time fluorescence reverse transcription quantitative PCR

The total RNA from CCM01, CCM01 + Tween 40, CCM 02, CCM02 + Tween 40, CCM03, and CCM03 + Tween 40 conditions after 48 h fermentation was extracted using a Trizol Plus RNA Purification Kit according to the manufacturer’s instructions (Invitrogen™, USA). cDNA was synthesized by the Primer Script™ RT Reagent Kit with gDNA Eraser (TaKaRa, Japan). The transcription levels of the *argB*, *zwf*, *odhA*, *lpdA*, *sucB*, *dtsR1*, *pknG*, and *ppp* genes were analyzed by the real-time fluorescence reverse transcription quantitative PCR (RT-qPCR) using SYBR^®^ Premix Tag™ (Tli RNaseH Plus) Kit (TaKaRa, Japan) with the corresponding primers (listed in Table 4). 16S rRNA was adopted as a reference gene. The thermal cycling conditions were 95 °C for 30 s, followed by 40 cycles of 95 °C for 5 s and 60 °C for 30 s. A control reaction without template was implemented to evaluate primer dimmer formation. The relative transcription level for each gene was calculated through the $2^{{ - \Delta \Delta {\text{C}}_{\text{t}} }}$ method [41]. All the data are presented as the means ± standard deviations in three independent experiments.

### Statistical analysis

The data were statistically compared using ANOVA, and significant differences were identified by Tukey-*t* test (*P* < 0.05). These analyses were carried out in GraphPad Prism 5.0 software (GraphPad software Inc, California, USA).

## References

[1] Alba-Roth J, MÜller OA, Schopohl J, Werder KV (1988). Arginine stimulates growth hormone secretion by suppressing endogenous somatostatin secretion. J Clin Endocrinol Metab.

[2] Thams P, Capito K (1999). l-Arginine stimulation of glucose-induced insulin secretion through membrane depolarization and independent of nitric oxide. Eur J Endocrinol.

[3] Davis S (1972). Plasma levels of prolactin, growth hormone, and insulin in sheep following the infusion of arginine, leucine and phenylalanine. Endocrinology.

[4] Bronte V, Zanovello P (2005). Regulation of immune responses by l-arginine metabolism. Nat Rev Immunol.

[5] Ignarro LJ, Cirino G, Casini A, Napoli C (1999). Nitric oxide as a signaling molecule in the vascular system: an overview. J Cardiovasc Pharmacol.

[6] Nakayama K, Yoshida H (1972). Fermentative production of l-arginine. Agric Biol Chem.

[7] Rahman MM, Qin ZQ, Dou W, Zhiming R, Xu Z (2013). Over-expression of NAD kinase in *Corynebacterium crenatum* and its impact on l-arginine biosynthesis. Trop J Pharm Res.

[8] Ginesy M, Belotserkovsky J, Enman J, Isaksson L, Rova U (2015). Metabolic engineering of *Escherichia coli* for enhanced arginine biosynthesis. Microb Cell Fact.

[9] Kisumi M, Kato J, Sugiura M, Chibata I (1971). Production of l-arginine by arginine hydroxamate-resistant mutants of *Bacillus subtilis*. Appl Microbiol.

[10] Yoshida H, Araki K, Nakayama K (1981). l-arginine production by arginine analog-resistant mutants of microorganisms. Agric Biol Chem.

[11] Xu M, Rao Z, Dou W, Yang J, Jin J, Xu Z (2012). Site-directed mutagenesis and feedback-resistant *N*-acetyl-l-glutamate kinase (NAGK) increase *Corynebacterium crenatum*l-arginine production. Amino Acids.

[12] Xu M, Rao Z, Yang J, Xia H, Dou W, Jin J (2012). Heterologous and homologous expression of the arginine biosynthetic *arg*C ~ H cluster from *Corynebacterium crenatum* for improvement of l-arginine production. J Ind Microbiol Biotechnol.

[13] Xu M, Rao Z, Yang J, Dou W, Xu Z (2013). The effect of a LYSE exporter overexpression on l-arginine production in *Corynebacterium crenatum*. Curr Microbiol.

[14] Park SH, Kim HU, Kim TY, Park JS, Kim S-S, Lee SY (2014). Metabolic engineering of *Corynebacterium glutamicum* for l-arginine production. Nat Commum.

[15] Shin JH, Lee SY (2014). Metabolic engineering of microorganisms for the production of l-arginine and its derivatives. Microb Cell Fact.

[16] Kimura E, Yagoshi C, Kawahara Y, Ohsumi T, Nakamatsu T, Tokuda H (1999). Glutamate overproduction in *Corynebacterium glutamicum* triggered by a decrease in the level of a complex comprising DtsR and a biotin-containing subunit. Biosci Biotechnol Biochem.

[17] Kimura E (2002). Triggering mechanism of l-glutamate overproduction by DtsR1 in coryneform bacteria. J Biosci Bioeng.

[18] Yao W, Deng X, Zhong H, Liu M, Zheng P, Sun Z (2009). Double deletion of *dtsR1* and *pyc* induce efficient l-glutamate overproduction in *Corynebacterium glutamicum*. J Ind Microbiol Biotechnol.

[19] Xu H, Dou W, Xu H, Zhang X, Rao Z, Shi Z (2009). A two-stage oxygen supply strategy for enhanced l-arginine production by *Corynebacterium crenatum* based on metabolic fluxes analysis. Biochem Eng J.

[20] Dou W, Xu M, Cai D, Zhang X, Rao Z, Xu Z (2011). Improvement of l-arginine production by overexpression of a bifunctional ornithine acetyltransferase in *Corynebacterium crenatum*. Appl Biochem Biotechnol.

[21] Shimizu H, Tanaka H, Nakato A, Nagahisa K, Kimura E, Shioya S (2003). Effects of the changes in enzyme activities on metabolic flux redistribution around the 2-oxoglutarate branch in glutamate production by *Corynebacterium glutamicum*. Bioprocess Biosyst Eng.

[22] Asakura Y, Kimura E, Usuda Y, Kawahara Y, Matsui K, Osumi T (2007). Altered metabolic flux due to deletion of *odhA* causes l-glutamate overproduction in *Corynebacterium glutamicum*. Appl Environ Microbiol.

[23] Nakamura J, Hirano S, Ito H, Wachi M (2007). Mutations of the *Corynebacterium glutamicum NCgl1221* gene, encoding a mechanosensitive channel homolog, induce l-glutamic acid production. Appl Environ Microbiol.

[24] Hashimoto K, Nakamura K, Kuroda T, Yabe I, Nakamatsu T, Kawasaki H (2010). The protein encoded by *NCgl1221* in *Corynebacterium glutamicum* functions as a mechanosensitive channel. Biosci Biotechnol Biochem.

[25] Kimura E, Abe C, Kawahara Y, Nakamatsu T, Tokuda H (1997). A *dtsR* gene-disrupted mutant of *Brevibacterium lactofermentum* requires fatty acids for growth and efficiently produces l-glutamate in the presence of an excess of biotin. Biochem Bioph Res Commun.

[26] Takeno S, Murata R, Kobayashi R, Mitsuhashi S, Ikeda M (2010). Engineering of *Corynebacterium glutamicum* with an NADPH-generating glycolytic pathway for l-lysine production. Appl Environ Microbiol.

[27] Lennen RM, Pfleger BF (2012). Engineering *Escherichia coli* to synthesize free fatty acids. Trends Biotechnol.

[28] Usuda Y, Tujimoto N, Abe C, Asakura Y, Kimura E, Kawahara Y (1996). Molecular cloning of the *Corynebacterium glutamicum* (‘*Brevibacterium lactofermentum*’AJ12036) *odhA* gene encoding a novel type of 2-oxoglutarate dehydrogenase. Microbiology.

[29] Schwinde JW, Hertz PF, Sahm H, Eikmanns BJ, Guyonvarch A (2001). Lipoamide dehydrogenase from *Corynebacterium glutamicum*: molecular and physiological analysis of the *lpd* gene and characterization of the enzyme. Microbiology.

[30] Krawczyk S, Raasch K, Schultz C, Hoffelder M, Eggeling L, Bott M (2010). The FHA domain of OdhI interacts with the carboxyterminal 2-oxoglutarate dehydrogenase domain of OdhA in *Corynebacterium glutamicum*. FEBS Lett.

[31] Raasch K, Bocola M, Labahn J, Leitner A, Eggeling L, Bott M (2014). Interaction of 2-oxoglutarate dehydrogenase OdhA with its inhibitor OdhI in *Corynebacterium glutamicum*: mutants and a model. J Biotechnol.

[32] Niebisch A, Kabus A, Schultz C, Weil B, Bott M (2006). Corynebacterial protein kinase G controls 2-oxoglutarate dehydrogenase activity via the phosphorylation status of the OdhI protein. J Biol Chem.

[33] Schultz C, Niebisch A, Schwaiger A, Viets U, Metzger S, Bramkamp M (2009). Genetic and biochemical analysis of the serine/threonine protein kinases PknA, PknB, PknG and PknL of *Corynebacterium glutamicum*: evidence for non-essentiality and for phosphorylation of OdhI and FtsZ by multiple kinases. Mol Microbiol.

[34] Schultz C, Niebisch A, Gebel L, Bott M (2007). Glutamate production by *Corynebacterium glutamicum*: dependence on the oxoglutarate dehydrogenase inhibitor protein OdhI and protein kinase PknG. Appl Microbiol Biotechnol.

[35] Liu Z, Chen L, Hao N, Xu L, Li Y, Yan M et al (2015) Expression of glucose-6-phosphate dehydrogenase and 6-phosphogluconate dehydrogenase improve l-citrulline biosynthesis in *argG*-deleted *Corynebacterium glutamicum*. In: Advances in applied biotechnology, vol 333. Springer, Berlin, pp 197–204

[36] Kataoka M, Hashimoto KI, Yoshida M, Nakamatsu T, Horinouchi S, Kawasaki H (2006). Gene expression of *Corynebacterium glutamicum* in response to the conditions inducing glutamate overproduction. Lett Appl Microbiol.

[37] Xu J, Xia X, Zhang J, Guo Y, Qian H, Zhang W (2014). A method for gene amplification and simultaneous deletion in *Corynebacterium glutamicum* genome without any genetic markers. Plasmid.

[38] Miller GL (1959). Use of dinitrosalicylic acid reagent for determination of reducing sugar. Anal Chem.

[39] Shiio I, Ujigawa-Takeda K (1980). Presence and regulation of α-ketoglutarate dehydrogenase complex in a glutamate-producing bacterium, *Brevibacterium flavum*. Agric Biol Chem.

[40] Kim J, Hirasawa T, Sato Y, Nagahisa K, Furusawa C, Shimizu H (2009). Effect of *odhA* overexpression and *odhA* antisense RNA expression on Tween-40-triggered glutamate production by *Corynebacterium glutamicum*. Appl Microbiol Biotechnol.

[41] Livak KJ, Schmittgen TD (2001). Analysis of relative gene expression data using real-time quantitative PCR and the 2(−Delta Delta C (T)). Methods.

